# Human cardiac tissue in a microperfusion chamber simulating extracorporeal circulation - ischemia and apoptosis studies

**DOI:** 10.1186/1749-8090-5-3

**Published:** 2010-01-18

**Authors:** Engin Usta, Mirijam Renovanz, Migdat Mustafi, Gerhard Ziemer, Hermann Aebert

**Affiliations:** 1 Department of Thoracic-, Cardiac- and Vascular Surgery, Tübingen University Hospital, Germany

## Abstract

**Background:**

After coronary artery bypass grafting ischemia/reperfusion injury inducing cardiomyocyte apoptosis may occur. This surgery-related inflammatory reaction appears to be of extreme complexity with regard to its molecular, cellular and tissue mechanisms and many studies have been performed on animal models. However, finding retrieved from animal studies were only partially confirmed in humans. To investigate this phenomenon and to evaluate possible therapies in vitro, adequate human cardiomyocyte models are required. We established a tissue model of human cardiomyocytes preserving the complex tissue environment. To our knowledge human cardiac tissue has not been investigated in an experimental setup mimicking extracorporeal circulation just in accordance to clinical routine, yet.

**Methods:**

Cardiac biopsies were retrieved from the right auricle of patients undergoing elective coronary artery bypass grafting before cardiopulmonary bypass. The extracorporeal circulation was simulated by submitting the biopsies to varied conditions simulating cardioplegia (cp) and reperfusion (rep) in a microperfusion chamber. Cp/rep time sets were 20/7, 40/13 and 60/20 min. For analyses of the calcium homoeostasis the fluorescent calcium ion indicator FURA-2 and for apoptosis detection PARP-1 cleavage immunostaining were employed. Further the anti-apoptotic effect of carvedilol [10 μM] was investigated by adding into the perfusate.

**Results:**

Viable cardiomyocytes presented an intact calcium homoeostasis under physiologic conditions. Following cardioplegia and reperfusion a time-dependent elevation of cytosolic calcium as a sign of disarrangement of the calcium homoeostasis occurred. PARP-1 cleavage also showed a time-dependence whereas reperfusion had the highest impact on apoptosis. Cardioplegia and carvedilol could reduce apoptosis significantly, lowering it between 60-70% (p < 0.05).

**Conclusions:**

Our human cardiac preparation served as a reliable cellular model tool to study apoptosis in vitro. Decisively cardiac tissue from the right auricle can be easily obtained at nearly every cardiac operation avoiding biopsying of the myocardium or even experiments on animals.

The apoptotic damage induced by the ischemia/reperfusion stimulus could be significantly reduced by the cold crystalloid cardioplegia. The additional treatment of cardiomyocytes with a non-selective β-blocker, carvedilol had even a significantly higher reduction of apoptotis.

## Introduction

Following extracorporeal circulation with cardioplegic cardiac arrest and reperfusion death or apoptosis of cardiomyocytes may occur [1,2]. Apoptosis is the ultimate result of convergence of multiple signaling pathways triggered by events such as nutrient and oxygen deprivation, intracellular calcium overload and excessive reactive oxygen species production [3]. In the setting of cardiac surgery these events can finally result in contractile dysfunction of the myocardium [4] and atrial fibrillation [5]. Apoptosis of cardiac non-myocytes also contributes to maladaptive remodelling and the transition to decompensated congestive heart failure [6]. Regarding this potentially impact of apoptosis on clinical outcomes, there is a demand for therapeutical strategies.

This surgery-related inflammatory reaction appears to be of extreme complexity with regard to its molecular, cellular and tissue mechanisms and many studies have been performed on animal models [7-9]. However, finding retrieved from animal studies were only partially confirmed in humans. To study the comparability with human tissue, we established an in vitro model using human cardiac tissue preserving the complex tissue milieu of the myocytes.

## Materials and methods

### Ethics declaration

The investigation conforms with the principles outlined in the Declaration of Helsinki. In addition, approval was granted by the Ethics Committee of the Faculty of Medicine of the Eberhard-Karls-University of Tübingen, Germany (approval reference number 183/2002 V).

### Patient characteristics

60 patients undergoing elective coronary artery bypass grafting were included in this study and gave informed consent before study entry. The mean age of the patients was 57 ± 6 (mean ± SEM), 58% of the patients were female.

### Cardiac tissue

Human tissue was retrieved from the auricle of the right atrium of patients before cardiopulmonary bypass and was processed immediately. Each biopsy was transmuraly divided with a scalpel in about 8 to 10 cubic pieces measuring approximately 500 μm. Cardiac specimens were randomly determined for incubation (incubation time 30 min) with the fluorescent dye FURA 2-AM for calcium analyses or for studies on apoptosis (described in the following sections). Cardiac specimens were outside the body before being mounted and tested in the chamber system for a maximum of 45 min, but during the incubation time the oxygen supply was maintained continuously.

### Chemicals and buffer solutions

The modified Krebs-Henseleit buffer (KH) consisted of 115 mM NaCl, 4.5 mM KCl, 1.18 mM MgCl_2_, 1.25 mM CaCl2, 1.23 mM NaH_2_PO_4_, 1.19 mM Na_2_SO_4_, 80 mM glucose, and 10 mM HEPES, pH adjusted to 7.4 at 37°C with NaOH. The Ca-free medium was the standard medium lacking CaCl_2 _and containing 0.5 mM EGTA.

### Cardioplegic solution

The cardioplegic solution was prepared on the basis of Ca-free Krebs-Henseleit buffer (KH) consisting of 115 mM NaCl, 4.5 mM KCl, 1.18 mM MgCl_2_, 0.5 mM EGTA, 1.23 mM NaH_2_PO_4_, 1.19 mM Na_2_SO_4_, 80 mM glucose, and 10 mM HEPES, pH adjusted to 7.4 at 37°C with NaOH.

For cardioplegia a solution containing 60 mmol K^+ ^was added in a 1:4 proportion to the Ca-free KH buffer, which was administered at 4°C, in analogy to blood cardioplegia regimen [10]. The resulting K^+ ^concentration in this mixture was 16.5 mM.

### Cell viability

The viability of cardiomyocytes was assessed by trypan blue exclusion before each experiment under a Nikon Labophot Y-2A epiflurescence microscope and a Nikon ×20 long-distance objective (Cf Plan ELWD, Nikon, Nippon Kokagu K.K., Tokyo, Japan). Only cardiac specimens consisting of ≥ 99% viable cardiomyocytes in the centre were included. Due to the preparation the section margins (cutting edges) of the cardiac specimens contained 5-10% non-viable cardiomyocytes. Therefore only central parts of the cardiac specimens were analyzed.

### Microperfusion chamber with physical adhesion of the cardiac specimens

**Figure 1:**
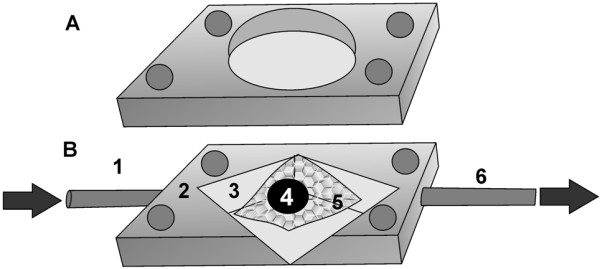
**Microperfusion chamber**. A: Top and B: Base Plexiglas components. Inlet pipe (1), Plexiglas block (2), rhomboid shaped chamber (3), cardiac tissue (4), nylon net (5) and outlet pipe (6). The top and base Plexiglas components were fastened with 4 screws.

Our self developed microperfusion chamber consisted of three components (Figure 1). A temperature-controlled plexiglas block was mounted on a metal sheet to allow fixation on the stage of a microscope. In the middle of this plexiglas block, a square, the size and depth of a coverglass, was cut out for insertion of a coverglass. The second component was mounted over the first, and consisted of another plexiglas block, however, with a rhomboid hole in the middle. In this free rhomboid chamber, a nylon net with a pore size of 400 μm was mounted diagonally. To enable perfusion of the rhomboid chamber, a thin pipe was introduced at one end of the block, entered the chamber and exited at the other end. This block was covered with a thin metal sheet with a coverglass in the middle of the sheet. A thin watertight silicon layer between each component sealed the microperfusion chamber.

The probe was fixed physically at the nylon net by the laminar flow of the hydrostatic perfusion system through the chamber. With a perfusion velocity of 5 ml/min, the probe was fixed to the net in a stable position, because of the laminar flow.

### Cardiomyocyte imaging

Calcium measurements were performed by digital imaging epifluorescence microscopy (Hamamatsu Photonics, Japan). A Nikon Labophot Y-2A epiflurescence microscope and a Nikon ×20 long-distance objective (Cf Plan ELWD, Nikon, Nippon Kokagu K.K., Tokyo, Japan) were used. Cardiac specimens were excited with a xenon arc lamp, and the emitted light was detected with a charge-coupled device camera (CCD camera). Image analysis was performed with the Hamamatsu Argus 50 system on a personal computer.

### Ratio imaging with FURA-2

FURA-2 is a Ca^2+ ^indicator. The AM ester is cleaved and hydrolyzed by non-specific esterases, resulting in the polyanionic indicator FURA-2, which leaks out of the cells far more slowly than its parent compound. This highly selective substance for calcium is nearly insensitive to slight fluctuations in the physiological range of the pH value. Fluorescence images of FURA-2 loaded cardiac specimens were obtained at excitation wavelengths of 340 nm and 380 nm, with an emission wavelength of 510 nm. Via FURA-2 the intracellular calcium concentration can be displayed by using ratio values 340/380. Ratio imaging [11] minimizes a number of negative effects which occur and disturb measurements like uneven dye loading, leakage of FURA-2 and bleaching. Background fluorescence determined in each experiment constituted to less than 5% of the fluorescence signal and therefore was subtracted from the intensities obtained at 340 and 380 nm.

### FURA-2 AM loading

Cardiac specimens were incubated with the fluorescent dye FURA-2 AM [100 μmol/l] (Molecular Probes, Eugene, OR, USA) for 30 min in 35°C KH (gassed with carbogen (95% O_2 _and 5% CO_2_)). Meanwhile the other half of the cardiac specimens were kept separately in 35°C KH (gassed with carbogen). At the end of the incubation the incubated and non-incubated cardiac specimens were transferred in 2 separate microperfusion chambers. After that the later decribed experimental protocol was carried out for both cardiac specimens simultaneously. The microperfusion chambers were arranged parallely so that same conditions like perfusion velocity, temperature and perfusion times were guaranteed.

### Calcium homoeostasis

For evaluation of the impact on the calcium homoeostasis in the cardiomyocytes the initial and final calcium ratio values had to be analyzed. This was defined as Δ-ratio; Δ-ratio = ratio_final_-ratio_initial_. Significant (p < 0.05) differences between both values were interpreted as calcium overload as a sign of disarranged calcium homoeostasis.

### Effect of carvedilol on apoptosis

Carvedilol is a nonspecific blocker that inhibits both β1- and β2-adrenergic receptors and furthermore is a strong antioxidant with antiapoptotic capacity [12]. To test whether treatment with a nonspecific β-blocker decreases apoptosis, we treated the cardiac specimens continuously with carvedilol. The administered concentration of carvedilol was 10 μmol/l. The cardiac specimens were subjected to various periods of cardioplegia (20, 40 or 60 min) followed by 1/3 of the chosen cardioplegia time as reperfusion (7, 13 or 20 min).

### Immunohistochemistry

The slides with the cryosections of the samples (10 μm) were processed prior to the staining according to the manufacturer's recommendation (Epitomics, Inc., Burlingame, CA, USA). The described chemicals were purchased from Biochrom, Berlin Germany. In brief, the cryosections were immersed into the staining dish containing the antigen retrieval solution: 9 ml of stock solution A (0.1 M citric acid solution) and 41 ml of stock solution B (0.1 M sodium citrate solution) were added to 450 ml of destillated H_2_O and adjusted to pH 6.0. After warming for 30 min in a rice cooker and cooling down the slides were washed with TBST (Tris-Buffered Saline and 0.1% Tween 20) for 5 min on a shaker. For the inactivation of endogenous peroxidases the slides were covered with 3% hydrogen peroxide for 10 min and later washed with TBST. After that the slides were immersed into the blocking solution (PBS (Dulbecco's Phosphate Buffered Salts) and 10% bovine serum albumin) for 1 hour.

Later the cryosections were incubated overnight in a humidified chamber (4°C) with antibodies against PARP-1 (Anti-Poly-(ADP-Ribose)-Polymerase)-cleavage (Epitomics, Inc., Burlingame, CA, USA). PARP is a zinc-dependent DNA binding protein that recognizes DNA strand breaks and is presumed to play a role in DNA repair. PARP is cleaved in vivo by caspase-3 [13]. The antibody only recognizes the p25 cleaved-form of PARP-1.

Later for detection to each section secondary HRP-conjugated anti-rabbit antibody (Epitomics, Inc., Burlingame, CA, USA) diluted in the blocking solution per manufacturer's recommendation was applied and incubated for 1 hour at room temperature.

The number of cells on the cryosections was determined by counting the nuclei of cardiomyocytes after staining with DAPI (4',6-Diamidino-2-phenylindole 2 HCl), a dye known to form fluorescent complexes with natural double-stranded DNA, under a fluorescence microscope (Zeiss, Jena, Germany). In each analysis three different areas of the cryosections were counted using 40-fold magnification. Fluorescence images (blue) of DAPI loaded cardiac specimens were obtained at an excitation wavelength of 360 nm, with an emission wavelength of 460 nm. DAPI was purchased from Sigma-Aldrich, Germany.

### Experimental protocol

The protocol was designed to simulate our clinical routine with the single difference that our cardioplegic solution with the same potassium concentration (16.5 mmol/l) and temperature (4°C) did not contain blood, but the Ca-free KH-buffer.

The 4 different groups (I-IV) were arranged as follows: cardioplegia (I) with or without reperfusion (II). The control groups receiving no cardioplegia were subjected to ischemia (III) with or without reperfusion (IV). All cardiac specimens had been prior incubated with the fluorescent dye FURA 2-AM for simultaneous calcium analyses. Each experiment, group (I-IV), was carried out with the specimens of one patient, i.e. specimens of patients were analyzed separately.

The cardiac specimens were initially equilibrated with KH for 5 min (32°C and gassed with carbogen. After that the cardioplegic solution (4°C) was administered for 5 min. For induction of apoptosis the cardiac specimens were subjected to various periods of cardioplegia (20, 40 or 60 min). During the cardioplegia period the perfusion of the microperfusion chamber was stopped, so that the oxygen supply was discontinued. Later reperfusion was initiated and it's duration was defined as being 1/3 of the chosen cardioplegia time (7, 13 or 20 min) just in analogy to our surgical routine. Reperfusion was modified so that initially for 2 min the cardiac specimens were reperfused with 35°C Ca-free KH and until the rest with 35°C KH. Otherwise the addition of calcium at this time would interfere with the calcium homoeostasis in the cardiomyocytes. So that discrimination between Ca-uptake induced fluorescence signal increase and reperfusion induced one would had been impossible.

Finally, the cardiac specimens were snap-frozen in liquid nitrogen.

### Statistical Analysis

Analysis of calcium recordings and graphics were performed using Sigma Plot software (version 9.0, SPSS Inc., Chicago, IL). Data are expressed as the mean ± standard error of mean (SEM) and statistical analysis was performed using the JMP software package (version 7.0, SAS Institute, Cary, NC, USA) employing multi-factorial analyses of variance tests (ANOVA) with Tukey's HSD post hoc test. A probability value of p < 0.05 was considered significant.

## Results

### Calcium analyses

**Figure 2:**
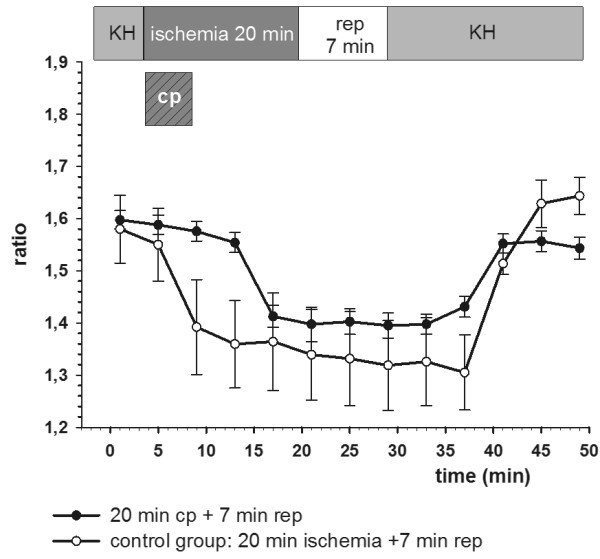
**Calcium homoeostasis was intact in the cardioplegia group**. In the control group (ischemia without cardioplegia) final calcium ratio values were significantly (p < 0.05) elevated. Ratio values are plotted as mean ± SEM of n = 5 experiments. Cp: cardioplegia for 5 min.

**Figure 3:**
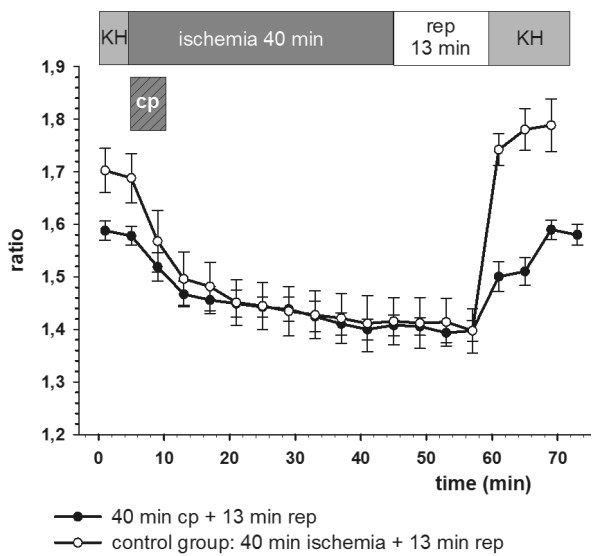
**Calcium homoeostasis was intact in the cardioplegia group**. In the control group (ischemia without cardioplegia) final calcium ratio values were significantly (p < 0.05) elevated. Ratio values are plotted as mean ± SEM of n = 5 experiments. Cp: cardioplegia for 5 min.

**Figure 4:**
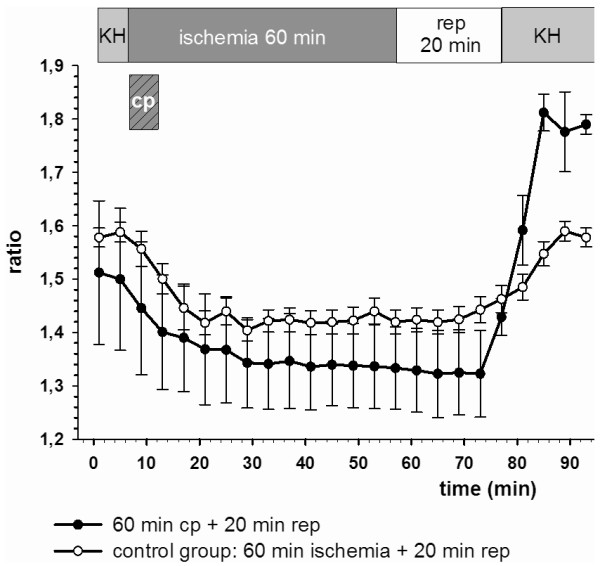
**Calcium homoeostasis was intact in the cardioplegia group**. In the control group (ischemia without cardioplegia) final calcium ratio values were significantly (p < 0.05) elevated. Ratio values are plotted as mean ± SEM of n = 5 experiments. Cp: cardioplegia for 5 min.

In the cardiac specimens treated with cardioplegia and reperfusion no significant (p < 0.05) cytosolic calcium increase and homoeostasis disarrangement could be detected (Table 1 and Figure 2, 3 and 4).

**Table 1:** Representing the effect of cardioplegia on calcium homoeostasis.

Group	Cp/rep in min	Ratio initial	Ratio final	Δ-ratio Ratio_final_-ratio_initial_	n =
**Control, non-cp + rep**	20/7	1.59 ± 0.04	1.65 ± 0.02	0.06 *	5

**Control, non-cp + rep**	40/13	1.68 ± 0.02	1.83 ± 0.02	0.12 *	5

**Control, non-cp + rep**	60/20	1.52 ± 0.01	1.64 ± 0.05	0.12 *	5

**Cp + rep**	20/7	1.57 ± 0.01	1.58 ± 0.01	0.01	5

**Cp + rep**	40/13	1.56 ± 0.02	1.56 ± 0.01	0	5

**Cp + rep**	60/20	1.60 ± 0.01	1.62 ± 0.01	0.02	5

Experimental protocol comparing calcium homoeostasis in the control group (non-cardioplegia and reperfusion) versus the cardioplegia and reperfusion group. Time sets were 20/7, 40/13 and 60/20 min. Δ-ratio was calculated as ratio_final_-ratio_initial_. Ratio values of n = 5 experiments are noted as mean ± SEM. Significant (p < 0.05) calcium homoeostasis disarrangements were detected in the control group (marked with stars).

In the control groups with non-cardioplegia and reperfusion ratio_final _was significantly (p < 0.05) higher than the ratio_initial _values, resulting in elevated Δ-ratio values correlating positively with the duration of ischemia and reperfusion times (Table 1 and Figures 2, 3 and 4).

### PARP-1 stained cardiomyocytes

**Figure 5:**
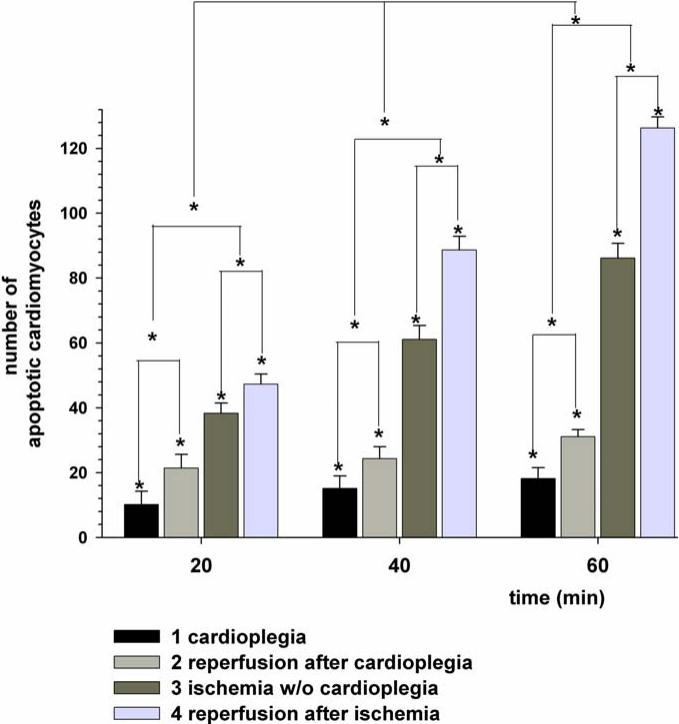
**Representative grayscaled fluorescent images of cardiomyocytes treated with cardioplegia and reperfusion (control group, first column) versus the same treatment plus the addition of carvedilol (second column)**. After DAPI counterstaining the greater nuclei of cardiomyocytes allow their distinction from fibroblasts with smaller nuclei. In PARP-1 cleavage positive, apoptotic cardiomyocytes nuclei feature an intensive granular fluorescence intensity (arrows). The exemplary images represent one experiment with the according cardioplegia and reperfusion time set noted on the left side. During the cryosection procedure artifacts presenting as nuclei conglomerates could not be avoided; these were excluded from analyses.

Apoptotic cardiomyocytes could be reliably distinguished of non-apoptotic ones. A bright nuclear staining, sometimes featuring granular structures was indicative for PARP-1 cleavage (Figure 5).

### The impact of cardioplegia on apoptosis

The mean total cardiomyocyte number in 3 analyzed central areas on the cryosections was 300 ± 25 (mean ± SEM). Usually the cryosections revealed around 21 ± 11 smaller or destructed nuclei, which were excluded.

**Figure 6:**
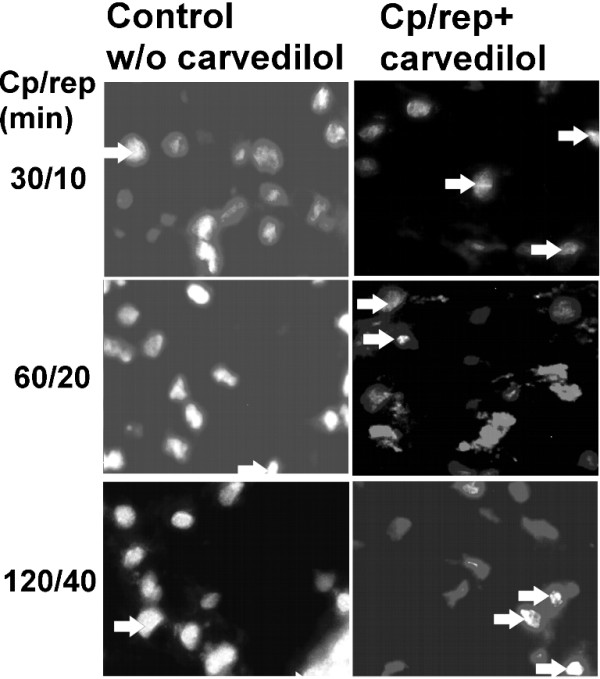
**Antiapoptotic effect of cardioplegia versus ischemia**. The x-axis represents time in min and the y-axis the number of PARP-1 cleavage positive, apoptotic cardiomyocytes. There is a time-dependent significant (p < 0.05) increase of apoptotic cardiomyocytes after reperfusion. Further there is a significant (p < 0.05) reduction of apoptosis in cardiomyocytes treated with cardioplegia versus ischemia. One column represents data of n = 5 experiments, noted as mean ± SEM. Stars mark the significances between the compared columns. The mean total cardiomyocyte number in 3 analyzed central areas on the cryosections was 300 ± 25 (mean ± SEM).

In general cardiomyocytes featured increasing PARP-1 expression depending on the duration of the ischemia and reperfusion period, just like in cardiomyocytes subjected to cardioplegia and reperfusion. The longer the cardioplegia and reperfusion periods lasted the higher was the number of PARP-1 positive or apoptotic cardiomyocytes. As presented in figure 6 cardioplegia could significantly (p < 0.05) reduce apoptosis compared to cardiomyocytes not subjected to cardioplegia.

### Effect of carvedilol on apoptotis

The longer the cardioplegia and reperfusion period lasted the higher was the percentage of PARP-1 cleavage positive or apoptotic cardiomyocytes. In contrast to non-treated cardiac specimens, sections prepared from cardiac specimens treated with carvedilol showed a significant (p < 0.05) reduction of PARP-1 cleavage positive cardiomyocytes (Figure 5).

## Discussion

In the present study our first goal was to apply cardioplegia and reperfusion just in accordance to our clinical routine to administer cardioplegia and reperfusion to simulate the extracorporeal circulation in our experimental model. Our second goal was to induce and detect apoptosis and concomitant alterations of the calcium homoeostasis in vitro in the presented experimental setup. Our third goal was to analyse the antiapoptotic properties of the non-selective β-blocker carvedilol. In our experimental model human cardiomyocytes were kept in their natural environment as intact cardiac tissue. Otherwise human papillary muscle could be employed but obtaining it before cardioplegic arrest is not an imaginable and feasible option during clinical routine. The simulation of ischemia in isolated cardiomyocyte models can provide important insights into the pathophysiology of myocardial ischemic injury and its underlying molecular mechanisms as was the subject in previous studies in isolated mammalian cardiomyocytes [7], isolated papillary muscle preparations [8] or animal heart models [9]. The distinctive difference of our study was to demonstrate our experimental set up utilizing the human atrial cardiac tissue model for apoptosis studies inducing apoptotis just in accordance to our clinical routine with cardioplegia and reperfusion without induction of simulated ischemia with N_2 _perfusion like in previous studies [14]. Like presented above the cardioplegia and reperfusion stimulus proved to be an adequate stimulus for apoptosis induction and is comparable with those in the literature [15,16].

Isolated cardiomyocyte models of simulated ischemia provided much insight into the pathophysiology of myocardial ischemic injury [17]. The use of isolated adult mammalian cardiomyocyte models can serve to discover underlying mechanisms occurring during ischemia versus those resulting from reperfusion. Results from several studies [18,19], however, indicate that reperfusion after myocardial ischemia can result in exacerbation of injury and apoptosis. Apoptosis is an important mechanism of active cellular death that is distinct from necrosis and has been implicated in the pathogenesis of a variety of degenerative and ischemic human diseases. In fact, several studies suggest that apoptosis is a reperfusion-related phenomenon [20]. Most of the studies were performed on isolated mammalian cardiomyocytes [21] or on isolated papillary muscle preparations [22] whereas the fewer human preparations were supposed to reflect the situation in the original enviroment better [23]. These techniques detect apoptosis at a very late stage, however for a better understanding of therapeutical manipulations earlier stages are warranted [24]. In the present study the usefullness of our atrial cardiac tissue model for apoptosis studies should be demonstrated. The atrial tissue is easily obtainable from patients undergoing open-heart surgery, it is simple to prepare, the procedure is inexpensive and human cardiac tissue is supposed to represent the original cellular environment better. Another advantage of the atrial tissue is that due to its morphology with a thin wall nutrition in vivo is mainly provided by diffusion.

In the present study human cardiomyocytes preserved in the natural cellular formation as atrial tissue preparation were submitted to varied cardioplegic-ischemia and reperfusion according to our routine cardioplegia regimen. The impact on apoptosis was investigated selectively after ischemia or reperfusion analysing cytosolic calcium changes and indicators for caspase-3 activation with resulting PARP cleavage. Viable, non-apoptotic cardiomyocytes were capable to maintain the essential calcium homoeostasis preventing a calcium overload. This was negatively influenced by longer duration of cardioplegia and reperfusion. One possible explanation for that could be uncontrolled calcium uptake per diffusion and release of the sarcoplasmatic reticulum. Hallmarks of apoptosis include morphological alterations such as cell shrinkage, membrane blebbing, chromatin condensation, and DNA fragmentation [15]. In contrast to that many apoptosis studies administered ischemia and reperfusion lasting many hours. If these experimental protocols were applied during routine cardiac surgery the result would be deleterious for the patients. As an indicator of initiation of apoptosis signal-pathways with resulting caspase activation [25] PARP cleavage was detectable in every experiment although reports about missing PARP cleavage exist [26]. For a definite statement further investigations especially on cardiomyocytes are necessary.

In our presented study the ischemia/reperfusion stimulus proved to be an adequate stimulus for apoptosis induction. Moreover, our results about the cardiomyocte apoptosis after an ischemia/reperfusion stimulus are comparable to those presented in the literature [15]. The partial inhibition of apoptosis by carvedilol as observed in our study has also been previously described in an animal model of end-stage heart failure [27,28]. A recent study could demonstrate that the cardioprotective effect of carvedilol against ischemia and/or reperfusion injury is via adenosine-dependent mechanisms [29]. The reason for partial suppression of apoptosis in the carvedilol treatment group in our study could also be dose related as being described in previous studies [30]. Carvedilol treatment inhibited apoptotis in a way that longer duration of cardioplegia and reperfusion had no significant increase of the apoptotis rate, whereas without carvedilol the apoptosis rate increased. The high apoptosis rate in the control group especially after 60 min cardioplegia and 20 min reperfusion should not be extrapolated into the in vivo situation without any caution as atrial and ventricular myocardium possess specific characteristics that may influence the susceptibility to ischaemia/reperfusion injury. One explanation is the reported difference in the distribution of potassium channels [31], which contribute to the characteristic differences between atrial and ventricular action potentials and may determine a different response to cardioplegia/reperfusion. Another explanation is that our experimental setup is distinctive of our surgical routine as we do not tolerate cardioplegia longer than 20 min and therefore apply cardioplegia in 20 min intervals. On the other hand even it is well known that carvedilol ameliorates cardiac ischaemic tissue injury [32], its antioxidant effects have not, to our knowledge, been reported in an experimental setup mimicking extracorporeal circulation, yet.

Conclusions of the present study are that our atrial tissue model is a reliable tool to investigate apoptosis in vitro. Decisively cardiac tissue from the right auricle can be easily obtained at nearly every cardiac operation avoiding biopsying of the myocardium or even experiments on animals. The ischemia/reperfusion stimulus induced apoptosis in cardiomyocytes depending on the duration of the stimulus. Apoptotic cardiomyocytes featured alterations of their calcium homeostatis resulting in a calcium overload. At the same time the apoptotic damage induced by the ischemia/reperfusion stimulus could be significantly reduced by the cold crystalloid cardioplegia. The additional treatment of cardiomyocytes with a non-selective β-blocker, carvedilol had even a significantly higher reduction of apoptotis.

Finally the relatively short cardioplegic-ischemia and reperfusion periods could be interpreted as limitation of the present study. Our future perspectives are to widen the research on other drugs with potential anti-apoptotic effect. Another limitation is that in this study only a single concentration of carvedilol was employed. Therefore, detailed dose-response relationships of carvedilol on apoptotic events have not been investigated. Nevertheless, with the concentration employed in this study, apoptotic events could be inhibited considerably. Furthermore the primary purpose of this study was to test the principle of β-blockade on apoptotic events rather than to study dose-response relationships. However, our results indicate a definite beneficial effect of carvedilol on apoptotic events at least in vitro.

## Competing interests

The authors declare that they have no competing interests.

## Authors' contributions

EU carried out the routine preoperative examinations, patient evaluation and participated in the study design and coordination. EU performed the statistical analysis. MR and MM participated in the experiments and data evaluation. HA and GZ conceived of the study, and participated in its design and coordination. All authors read and approved the final manuscript.

## Acknowledgements

This work was supported by a research grant (Fortüne 1232126.2) of the Faculty of Medicine of the Eberhard-Karls-University Tübingen, Germany.

We thank Dietz K., M.D., former head of the department of Medical Statistics, Eberhard-Karls-University Tübingen for performing the statistical analyses.
